# Differences related to sex in adolescent obese Zucker rats: locomotor activity and dendritic arborization of neurons in layer V of the primary motor cortex

**DOI:** 10.1007/s00418-026-02537-y

**Published:** 2026-09-06

**Authors:** Lesly Domínguez-Juárez, Adriana Berenice Silva-Gómez

**Affiliations:** Laboratorio de Neurofisiología Experimental, Facultad de Ciencias Biológicas, Benemérita Universidad Autónoma de Puebla, Puebla, México

**Keywords:** Primary motor cortex, Obese Zucker rats, Adolescent rats, Dendritic spines, Dendritic arborization

## Abstract

Obesity is a metabolic disorder characterized by the accumulation of excess body fat, which has been linked to skeletal muscle and motor behavior changes. Despite the high prevalence of obesity among children and adolescents in Mexico, it has mostly been studied using adult obese animal models. The objective of the present study was to analyze open-field locomotor activity and dendritic length and dendritic spine density in layer V pyramidal neurons of the primary motor cortex (M1) in adolescent (60-day-old) obese Zucker diabetic fatty (ZDF) rats of both sexes. Long-Evans (LE) rats were used as the control group. Female ZDF rats exhibited increased body weight, Lee index, and abdominal circumference. In contrast, male ZDF rats showed a shorter body length together with increased Lee index and abdominal circumference. However, ZDF rats of both sexes displayed elevated triglyceride and cholesterol levels. In addition, hyperglycemia was observed in male ZDF rats. Female ZDF rats showed decreased exploratory activity. The dendritic length of the basilar arbor of M1 neurons was significantly shorter in female and male ZDF rats, with ZDF males exhibiting a smaller basilar arbor than ZDF females. This last result was also observed in the apical arbor of these neurons. There was also a decrease in basilar and apical dendritic spine density in ZDF rats of both sexes. Our results indicate that female ZDF rats show alterations in exploratory behavior and dendritic arborization, whereas ZDF males are affected in terms of dendritic arborization in M1 neurons, which suggests sex differences in the effects of obesity in adolescent Zucker rats.

## Introduction

Obesity is a complex, multifactorial, and chronic condition characterized by the accumulation of excess body fat. It has a high prevalence worldwide and has been linked to a reduced life expectancy, since obese individuals have a higher chance of developing cardiovascular diseases, metabolic disorders, and dementia (Tomassoni et al. 2020; Sui et al. 2020). In 2022, the World Health Organization (WHO) reported that approximately 160 million individuals aged 5–19 years were living with obesity (World Health Organization 2025). Despite this high prevalence, most experimental studies on obesity continue to rely on adult animal models, such as the Zucker diabetic fatty (ZDF) rat, to investigate the effects of this condition on a wide range of biological processes (Aleixandre de Artiñano and Miguel Castro 2009; Al-war et al. 2016).

The ZDF rat originated from crosses between Merck stock M (13 M strain) and Sherman rats (Zucker and Zucker 1961) and carries an autosomal recessive mutation in the leptin receptor gene (Chua et al. 1996). This mutation causes obesity between the third and fifth weeks of life (Guzzardi et al. 2022), hyperglycemia, and dyslipidemias (Al-war et al. 2016; Clark et al. 1983; Raut and Bandawane 2018), among other metabolic disorders.

The ZDF rat has been widely used to study metabolic disorders; however, relatively little information has been generated regarding the consequences of obesity on the structure and function of the central nervous system (CNS). Moreover, these studies have been conducted predominantly in male rats. In this regard, recent evidence has shown that male ZDF rats exhibit reduced dendritic arborization in neurons of the hippocampus, dentate gyrus, and prefrontal cortex (Manuel-Sánchez et al. 2022; Utrilla-Ramos et al. 2024). However, no information is currently available regarding these brain structures in female ZDF rats. Likewise, female ZDF rats have been reported to exhibit reduced dendritic length in neurons of the ventromedial hypothalamic nucleus (Bravo-Durán et al. 2021), a brain region that has not yet been investigated in male ZDF rats. Therefore, considering the possibility of natural sex-related differences, the inclusion of animals of both sexes was of particular interest in the present study. Specifically, the present study aimed to evaluate the dendritic arborization of layer V pyramidal neurons in the primary motor cortex (M1) of adolescent ZDF rats. This objective was based on previous reports showing that 10-week-old male ZDF rats exhibit reduced locomotor activity, reflected by a shorter distance traveled and lower locomotor speed (Stern and Johnson 1977), as well as reduced grip strength. These findings suggest the presence of obesity-related alterations at the level of the central nervous system (Mzhelskaya et al. 2020).

In general, the M1 is recognized for its ability to modulate movement, motor control, and the learning of novel movement sequences (Gloor et al. 2015) through its complex connectivity with subcortical nuclei (globus pallidus, central amygdala, and dorsal raphe nucleus, among others) and thalamo-motor and frontal cortical areas (Lee et al. 2022). The M1 is responsible for pushing, throwing, holding, and gripping movements, as well as pre- and post-movement (Papale and Hooks 2018; Martín-Vázquez et al. 2018); it contains pyramidal neurons of highly variable morphology (Gao and Zheng 2004) whose dendritic arbors have thus been divided into apical, oblique apical, and basal parts (Akulinin et al. 1997). The arbors of these neurons are specialized in receiving information via dendritic spines (Davidson et al. 2020), which are structures that process and integrate information received from other neurons and exhibit a highly dynamic plastic activity (Vázquez-Hernández et al. 2017). Pyramidal neurons in layer V of the M1 integrate excitatory and inhibitory information across the motor cortex; they are the main excitatory input to the striatum involved in the corticostriatal pathway for motor control, and the efficiency of synaptic transmissions can be altered by motor experience (Pérez-Sisqués et al. 2021).

Specifically, during exploratory behavior, the M1 enhances the ability of subcortical motor systems to respond flexibly to unexpected situations by conferring robustness and adaptability to motor behavior (Lopes et al. 2023). This cortical region undergoes substantial structural and functional changes that support motor learning (Peters et al. 2017), particularly during puberty, a critical developmental period, especially in females (Juraska and Drzewiecki 2020), when synaptic pruning reshapes dendritic spine populations (Tekin et al. 2024).

Nevertheless, during adolescence in rats (postnatal days 31–60; Juraska and Drzewiecki 2020), layer V neurons of the M1 have already attained a structural and functional organization that closely resembles the adult phenotype. Although subtle refinements in electrophysiological activity continue to occur (Benedetti et al. 2020), structural changes during this period appear to be primarily driven by motor learning.

## Materials and methods

### Biological material

Sixty-day-old rats, considered adolescents according to Sengupta (2013), were used for the experiments: 20 (10 females and 10 males) of the Long-Evans strain (LE) as a control group and 20 (10 females and 10 males) of the ZDF strain as an experimental group. The sample size was determined to detect differences between groups with a statistical power of 80% and a significance level of *α* = 0.05, taking into account effect sizes reported in a previous study (Silva-Gómez et al. 2003). The rats were obtained from the Claude Bernard Animal Facility of the Benemérita Universidad Autónoma de Puebla (BUAP), where they were kept under controlled conditions at 21 ± 1 °C, 55% relative humidity, and a 12:12 light–dark cycle, with food and water ad libitum. All procedures in the present study were carried out in accordance with the technical standards for the care and use of laboratory animals established by the Official Mexican Standard NOM-062-ZOO-1999 and the Institutional Committee for the Care and Use of Laboratory Animals (CICUAL) of BUAP with the approved project no. 100274211-UALVIEP-22/2. The study was also conducted in accordance with the Animals in Research: Reporting In Vivo Experiments (ARRIVE) guidelines (Kilkenny et al. 2010).

### Open-field locomotor activity

In the open-field test, an animal is exposed to an open environment without an external stimulus and allowed to explore for a specific period of time to evaluate locomotor and exploratory activity. The open-field arena can be circular, rectangular, or square (Quillfeldt 2015). In the present study, 59-day-old rats from each group were individually placed in a 60 × 60 × 60 cm wooden box for 15 min. Each trial was video recorded for subsequent analysis of the number of square entries, number of corner entries, and time spent in the corners. Behavioral data were analyzed using a custom developed .cpp software program designed in our laboratory to quantify the frequency and duration of the different behavioral patterns. Entry into a square was defined as the placement of all four paws within the square. These data were used to calculate distance traveled during the trial. The box was cleaned before and after each trial to prevent olfactory cues from influencing the animals’ behavior.

### Zoometric parameters

At 60 days of age, the rats were anesthetized with a xylazine (10 mg/kg/ml; Cheminova de México, S.A. de C.V.) and ketamine (75 mg/kg/ml; Wildlife Pharmaceuticals México S.A. de C.V.) solution (i.p.). Once the rats were deeply sedated, body size (naso-anal length, cm) and abdominal circumference (cm) were measured in the supine position. Data of body weight (g) and body size were used to calculate the Lee index, where values ≥ 0.310 indicate obesity (Lee 1929; Novelli et al. 2007; Arika et al. 2019).

### Lipid and glucose concentrations

Immediately after obtaining the body measurements, 2 ml of blood were collected by cardiac puncture and centrifuged at 1700 g for 15 min to obtain the plasma. The following concentrations were quantified: (1) triglycerides, using the glycerol phosphate oxidase–phenol aminophenazone peroxidase (GPO-PAP) triglyceride kit (cat. 8,001,902); (2) cholesterol, with the cholesterol oxidase (CHOD)-PAP total cholesterol kit (cat. 8,001,209); and (3) glucose, with the glucose oxidase peroxidase (GOP)-PAP glucose kit (cat. 8001502). The kits were purchased through Corporativo Grupo Mexlab, S.A. de C.V., Mexico (https://www.grupomexlab.com).

### Golgi–Cox method

**Fig. 1:**
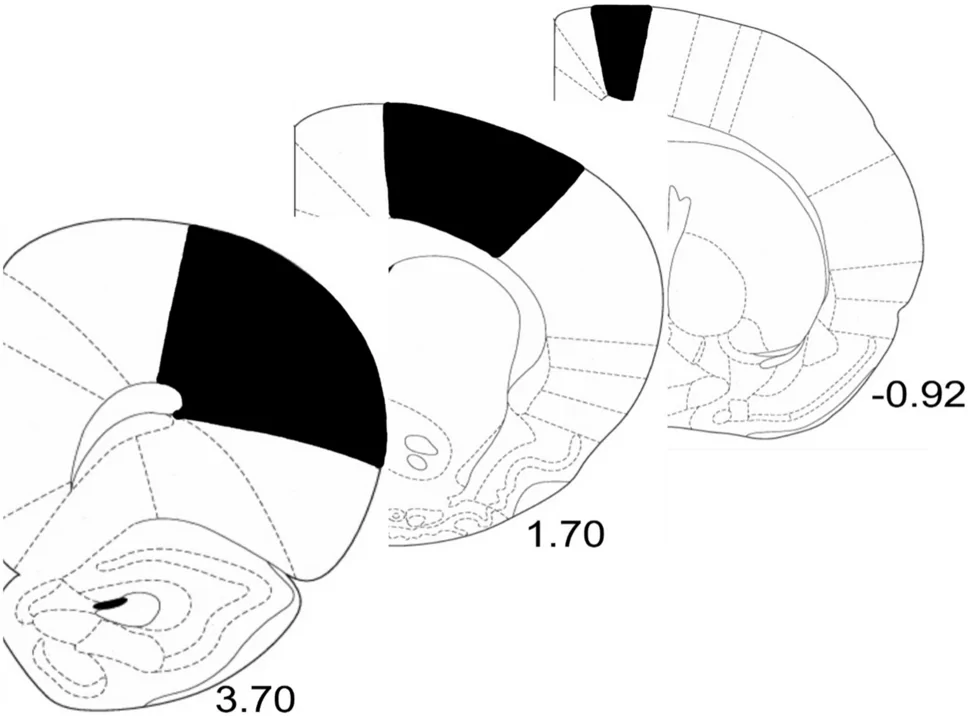
Location of the M1 in different coronal sections of the rat brain. Bregma coordinates (mm) taken and modified from Paxinos and Watson (1998)

Immediately after collecting the blood, the animals were intracardially perfused with 100 ml of 0.9% saline solution (Merck, México) to obtain blood-free brains, which were removed by craniotomy. The brains were stored in 20 ml of Golgi–Cox solution (potassium dichromate, 5% solution; mercuric chloride, 5% solution; potassium chromate, 5% solution, in distilled water (Raju et al. 2004); Reactivos Química Meyer) for 14 days in complete darkness. After this time, the solution was replaced with 30% sucrose (Difco Laboratories, USA), in which the brains were kept for 3–5 days. Subsequently, a semiautomatic vibratome (Leica VT1000 S) was used to obtain coronal sections of 200 µm in thickness at the level of the M1 at 3.70 to −0.92 mm relative to Bregma (Fig. 1; Paxinos and Watson 1998).

The histological sections were mounted on 2% gelatinized slides and incubated in a humidity chamber overnight. The next day, the slides were washed with distilled water for 1 min, incubated in ammonium hydroxide (28%) (Golden Bell, México) for 30 min, and then washed again with distilled water for 1 min. The slides were subsequently immersed in Kodak rapid fixer for another 30 min. Immediately afterwards, the tissue was washed again with distilled water for 1 min. After the developing step, the tissue was dehydrated through increasing alcohol concentrations (50%, 70%, 90%, and 95%) (Reactivos Química Meyer, México) for 1 min each and bathed twice in 100% alcohol for 5 min each time. Finally, the tissue was immersed in xylol for 15 min and mounted using synthetic resin medium Entellan (Gibb and Kolb 1998; Das et al. 2013).

### Dendritic arborization and spine density

Of the 13–14 coronal sections obtained from each brain, the search for layer V pyramidal neurons in the M1 was conducted in sections 6–9 (2.50–1.90 mm relative to Bregma). The criteria used for neuronal selection were as follows: (1) neurons relatively isolated from neighboring cells, allowing for clear visualization of their dendritic arbors without overlap; (2) pyramidal neurons with the soma located in layer V of M1; (3) neurons with a triangular-shaped soma; (4) neurons displaying both a clearly identifiable basilar dendritic arbor, emerging from the base of the soma, and an apical dendritic arbor, arising from the apex of the soma; (5) neurons with intact dendritic trees and no truncated dendrites; (6) neurons with at least three primary basilar dendrites, each exhibiting at least one branch point; (7) neurons showing clear and complete cellular staining; and (8) neurons exhibiting dendritic spines on both the apical and basilar dendritic arbors (Silva-Gómez et al. 2003). Two-dimensional reconstructions of ten neurons, five from the right hemisphere and five from the left hemisphere (× 400), and of dendritic segments of third-order branches (× 1000), were obtained using a Leica DM1000 light-emitting diode (LED) optical microscope equipped with a drawing arm or camera lucida. Dendritic spine density was quantified using drawings of dendritic segments measuring at least 10 µm in length (Gibb and Kolb 1998). A transparent sheet with concentric circles was placed on the drawings of the neurons to perform a Sholl analysis (1953) and quantify dendritic arborization by counting the number of dendritic intersections at each concentric circle. The basilar and apical arbors of the neurons were analyzed separately, and the data were used to calculate dendritic length by branch order and total dendritic length.

Data collection was performed by a rigorously trained experimenter who strictly adhered to the predefined inclusion criteria for selecting the neurons included in the study. In addition, under close supervision, the data analysis protocol was applied consistently and uniformly across all samples, thereby minimizing potential sources of bias and ensuring the reliability of the results.

### Statistical analysis

The obtained data showed a normal distribution, as indicated by a Shapiro–Wilk test, and were analyzed with a two-way analysis of variance (ANOVA), followed by Tukey’s multiple comparison test, where strain and sex were the independent variables. Differences were considered significant when *P* < 0.05. GraphPad Prism software, version 11.0.2 (100), was used for statistical analyses and graph preparation.

## Results

To simplify the graphical presentation of the results, only statistically significant differences are indicated in the figures, using one asterisk (^*^) to denote a significance level of *P* < 0.05 and two asterisks (^**^) to denote *P* < 0.001. However, the specific values obtained from each statistical analysis performed are provided in the corresponding tables.

### Zoometric parameters

**Fig. 2:**
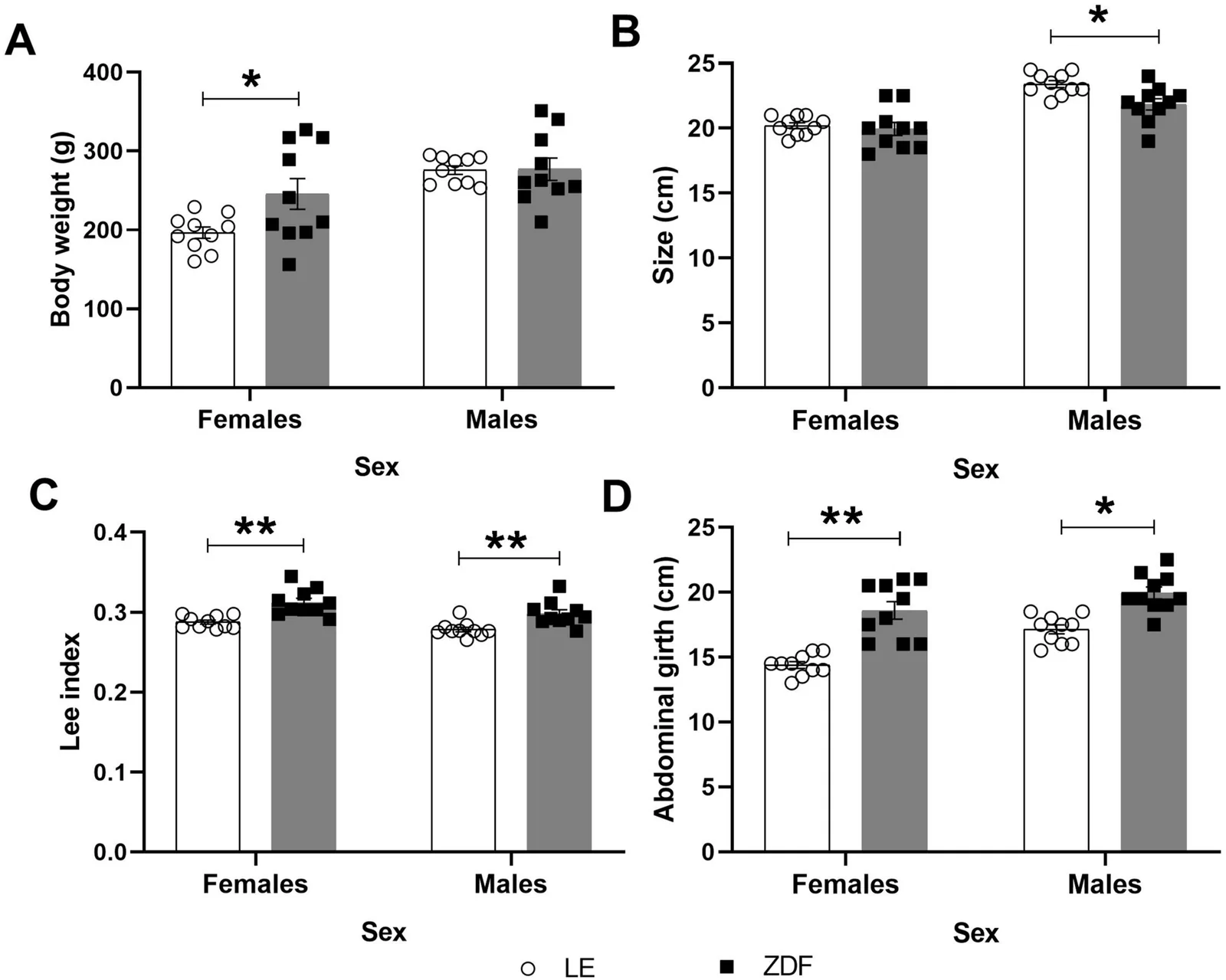
Zoometric measurements of female and male LE and ZDF rats. **(A)** ZDF females were significantly heavier, whereas ZDF males did not show changes in weight. **(B)** ZDF males were significantly smaller than LE males. **(C)** Both ZDF females and males showed higher Lee index values. **(D)** Both ZDF females and males had significantly greater abdominal circumference. The values shown are the mean ± standard error of the mean (SEM). *N* = 10 per group. ^*^P < 0.05, ^**^P < 0.001

Female ZDF rats were significantly heavier (245.700 ± 19.532 g) than female LE rats (196.600 ± 7.142 g), whereas no significant difference in body weight was found between male ZDF (277.100 ± 14.209 g) and male LE rats (275.800 ± 5.409 g; Fig. 2A; Table 1, row A). With respect to body size, there was no significant difference between female ZDF (19.950 ± 0.497 cm) and female LE rats (20.200 ± 0.226 cm), whereas male ZDF rats were smaller (19.950 ± 0.407 cm) than male LE rats (23.400 ± 0.267 cm; Fig. 2B; Table 1, row B). Unlike these two variables, both female and male ZDF rats showed significantly higher Lee index values compared with their respective control groups (ZDF females 0.312 ± 0.016 versus LE females 0.288 ± 0.008; ZDF males 0.298 ± 0.015 versus LE males 0.278 ± 0.009; Fig. 2C; Table 1, row C). The same result was obtained for abdominal circumference (ZDF females 18.600 ± 0.678 cm versus LE females 14.400 ± 0.256 cm; ZDF males 19.950 ± 0.456 cm versus LE males 17.150 ± 0.342 cm; Fig. 2D; Table 1, row D).

**Table 1:** Statistical data from the two-way ANOVA applied to the zoometric parameters

		Interaction			Sex			Strain		
	Variable	F_(1, 36)_	*P*	Total variation (%)	F_(1, 36)_	*P*	Total variation (%)	F_(1, 36)_	*P*	Total variation (%)
A	Body weight (g)	3.443	0.0717	5.580	18.43	< 0.0001	29.87	3.828	0.3828	6.203
B	Body size (cm)	3.027	0.0904	3.311	46.59	< 0.0001	50.96	5.803	0.0212	6.348
C	Lee index	0.3593	0.5526	0.4764	8.396	0.0064	11.13	30.67	< 0.0001	40.66
D	Abdominal circumference (cm)	2.304	0.1377	1.992	19.76	< 0.0001	17.09	57.61	< 0.0001	49.80

### Lipid and glucose concentrations

**Fig. 3:**
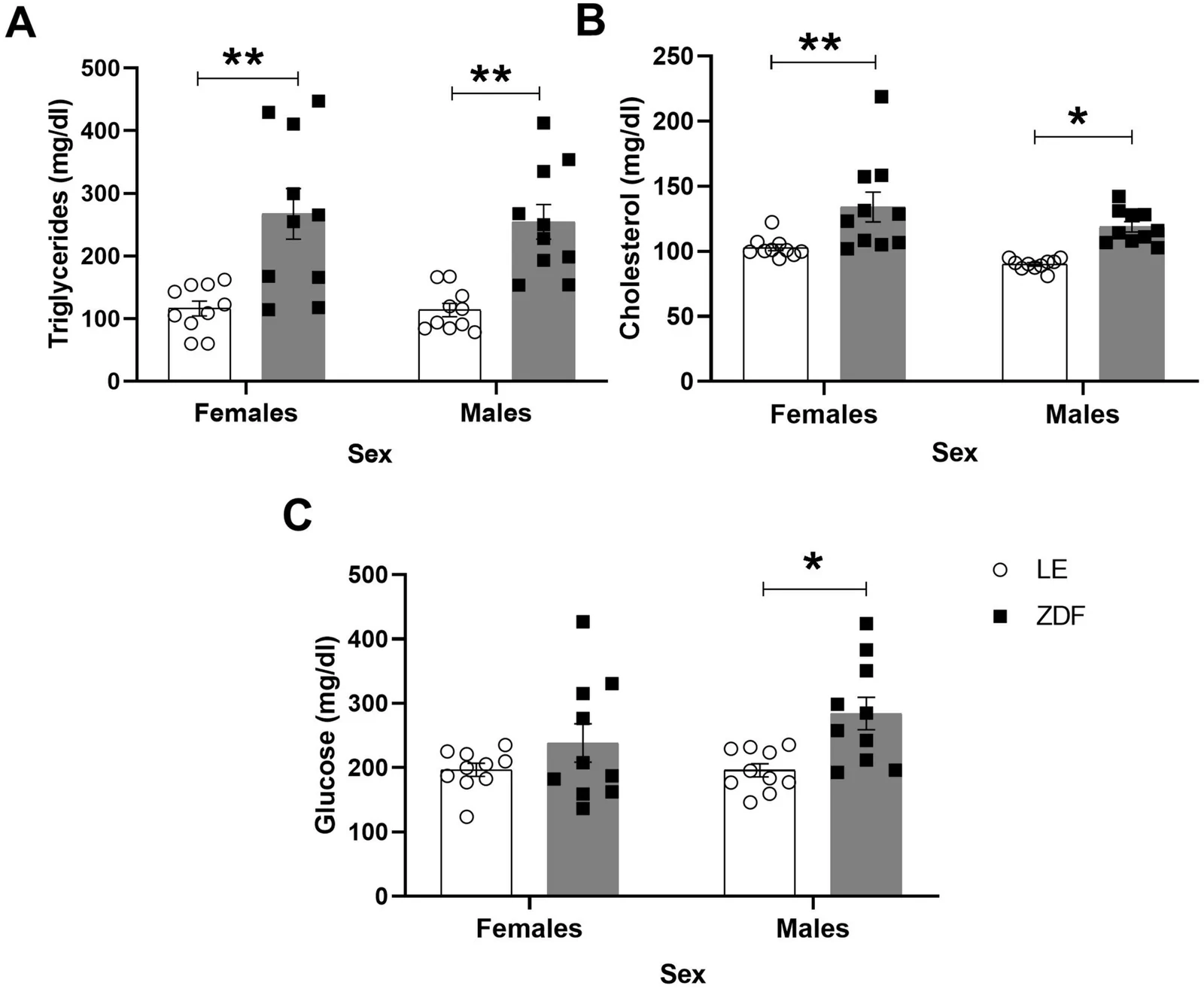
Plasma lipid and glucose concentrations in female and male ZDF rats. **(A)** There was an increase in triglyceride and **(B)** cholesterol levels in both female and male ZDF rats. **(C)** There was a significant increase in glucose concentration in male ZDF rats compared with their respective control group. Values shown are the mean ± SEM. *N* = 10 per group. ^*^P < 0.05, ^**^P < 0.001

Triglyceride levels were significantly higher in female and male ZDF rats compared with their respective LE control groups (ZDF females 267.288 ± 40.317 mg/dl versus LE females 116.397 ± 11.933 mg/dl and ZDF males 254.648 ± 27.758 mg/dl versus LE males 113.805 ± 10.548 mg/dl; Fig. 3A; Table 2, row A). Furthermore, significantly higher total cholesterol levels were observed in both female (134.046 ± 29.416 mg/dl versus LE females 102.887 ± 2.460 mg/dl) and male ZDF rats (118.853 ± 4.061 mg/dl versus LE males 90.033 ± 1.313 mg/dl; Fig. 3B; Table 2, row B) compared with their respective control groups. In the case of glucose levels, male ZDF rats showed higher values (284.219 ± 25.347 mg/dl versus LE males 195.940 ± 10.231 mg/dl), while female ZDF rats showed no changes in this variable (238.404 ± 29.909 mg/dl versus LE females 196.661 ± 10.073 mg/dl; Fig. 3C; Table 2, row C).

**Table 2:** Statistical data from the two-way ANOVA applied to the plasma parameters

		Interaction			Sex			Strain		
	Variable	F_(1, 36)_	*P*	Total variation (%)	F_(1, 36)_	*P*	Total variation (%)	F_(1, 36)_	*P*	Total variation (%)
A	Triglyceride (mg/dl)	0.03810	0.8463	0.5583	0.08757	0.7690	0.1283	32.12	< 0.0001	47.07
B	Cholesterol (mg/dl)	0.03541	0.8518	0.05499	5.089	0.0303	7.902	23.27	< 0.0001	36.14
C	Glucose (mg/dl)	1.242	0.2724	2.582	1.167	0.2873	2.425	9.699	0.0036	20.16

### Open-field locomotor activity

**Fig. 4:**
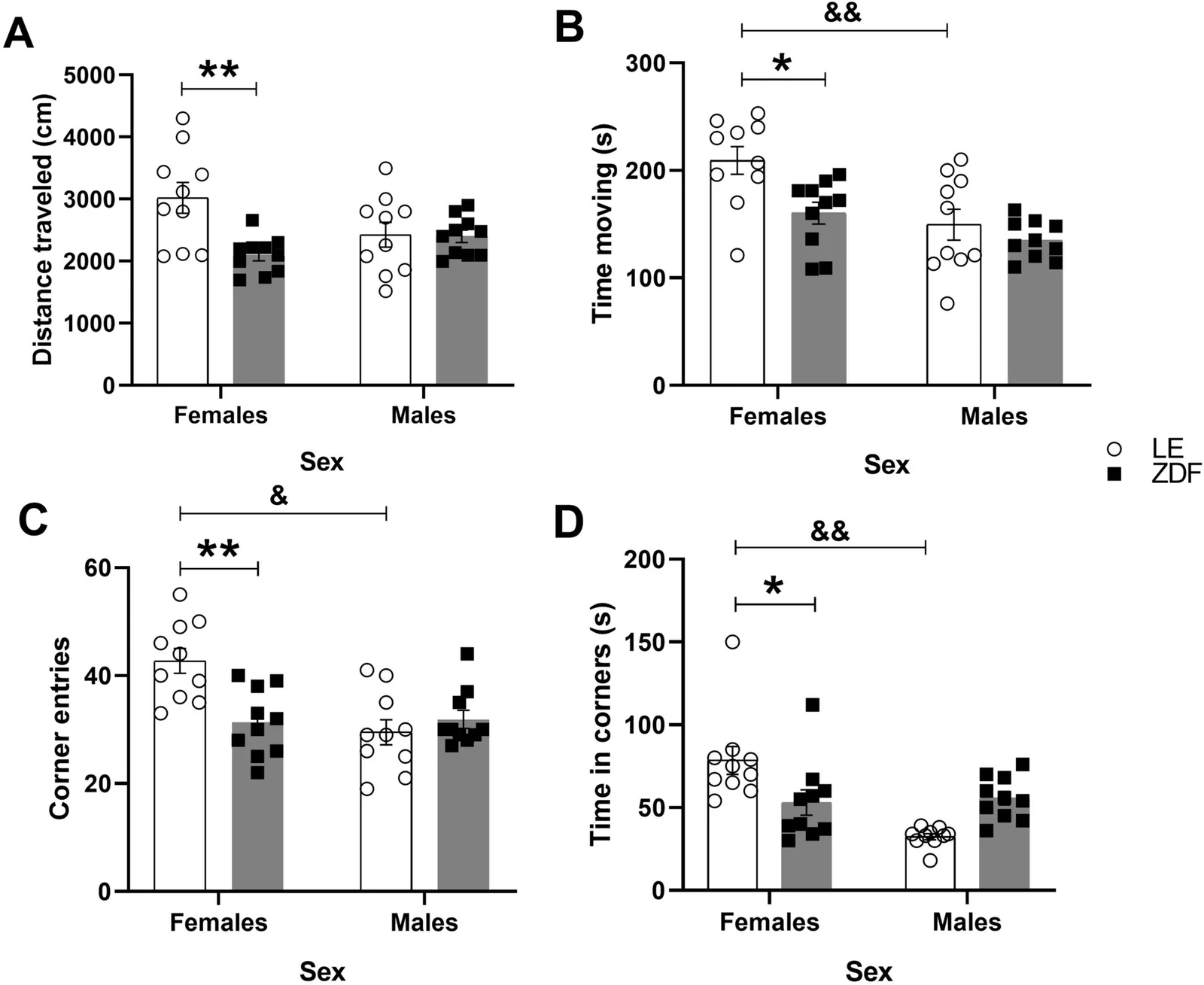
Exploratory activity in the open field. **(A)** Female ZDF rats traveled a shorter distance, **(B)** spent less time moving, **(C)** showed fewer corner entries, and **(D)** spent less time in the corners. In addition, female LE rats showed greater exploratory activity compared with male LE rats. The values shown are the mean ± SEM. *N* = 10 per group. ^*^P < 0.05, ^**^P < 0.001

In the open-field test, female ZDF rats displayed lower locomotor and exploratory activity than female LE rats, as evidenced by a shorter distance traveled (Fig. 4A; Table 3, row A), reduced time spent moving (Fig. 4B; Table 3, row B), fewer corner entries (Fig. 4C; Table 3, row C), and less time spent in the corners (Fig. 4D; Table 3, row D). In contrast, no significant differences were detected between male ZDF and male LE rats.

**Table 3:** Statistical data from the two-way ANOVA applied to the open-field locomotor activity parameters

		Interaction			Sex			Strain		
	Variable	F_(1, 36)_	*P*	Total variation (%)	F_(1, 36)_	*P*	Total variation (%)	F_(1, 36)_	*P*	Total variation (%)
A	Distance traveled (cm)	6.719	0.0137	13.19	0.6942	0.4102	1.363	7.521	0.0094	4.77
B	Time moving (s)	2.343	0.1346	3.866	14.30	0.0006	23.60	7.958	0.0077	13.13
C	Corner entries (number)	10.96	0.0021	18.04	9.138	0.0046	15.04	4.662	0.0376	7.673
D	Time in corners (s)	15.73	0.0003	24.46	12.55	0.0011	19.51	0.02924	0.8652	0.0454

### Dendritic length by branch order in M1 pyramidal neurons

**Fig. 5:**
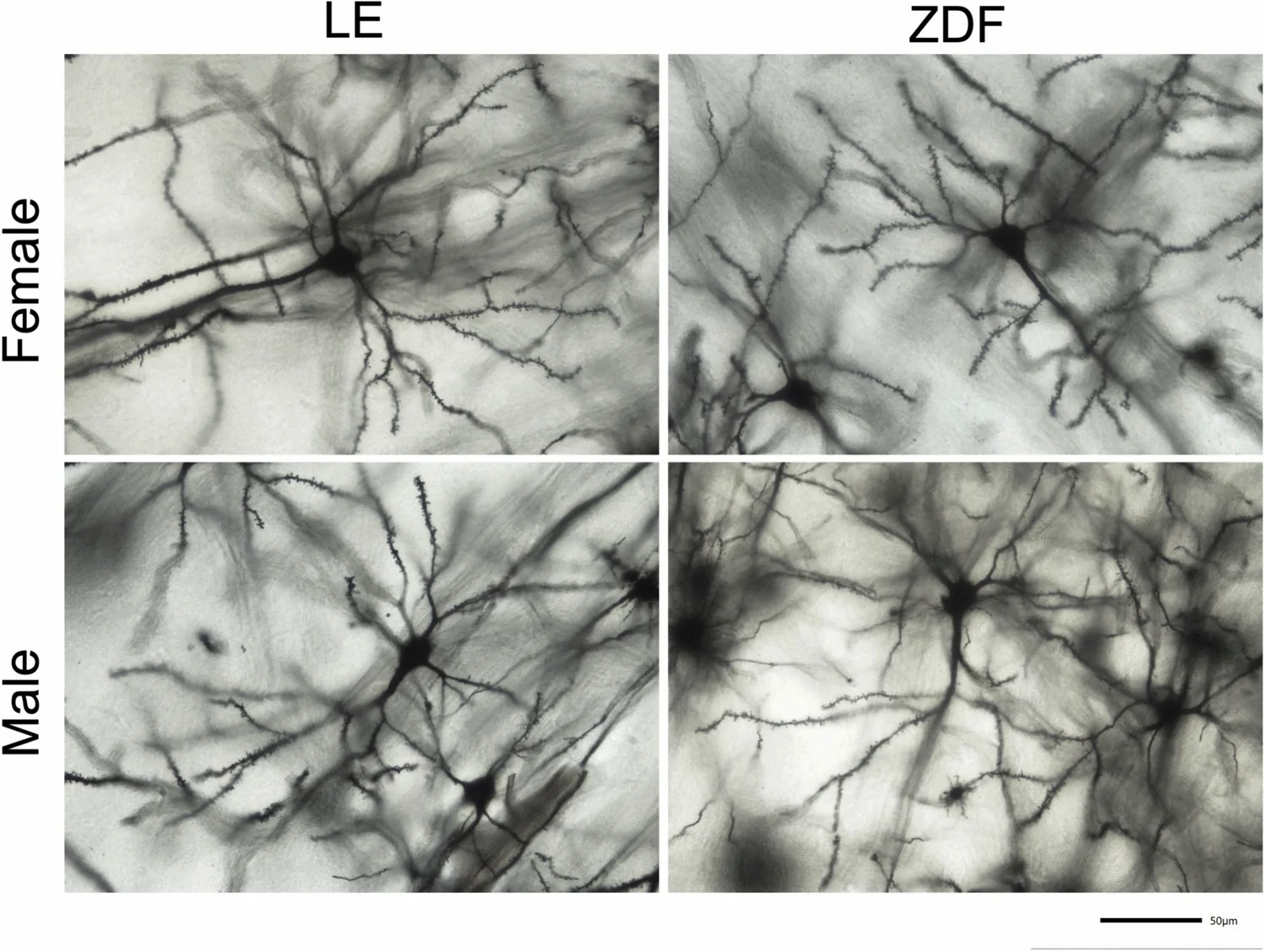
Example of neurons in M1 in female and male rats. Photomicrographs illustrating Golgi–Cox impregnation

**Fig. 6:**
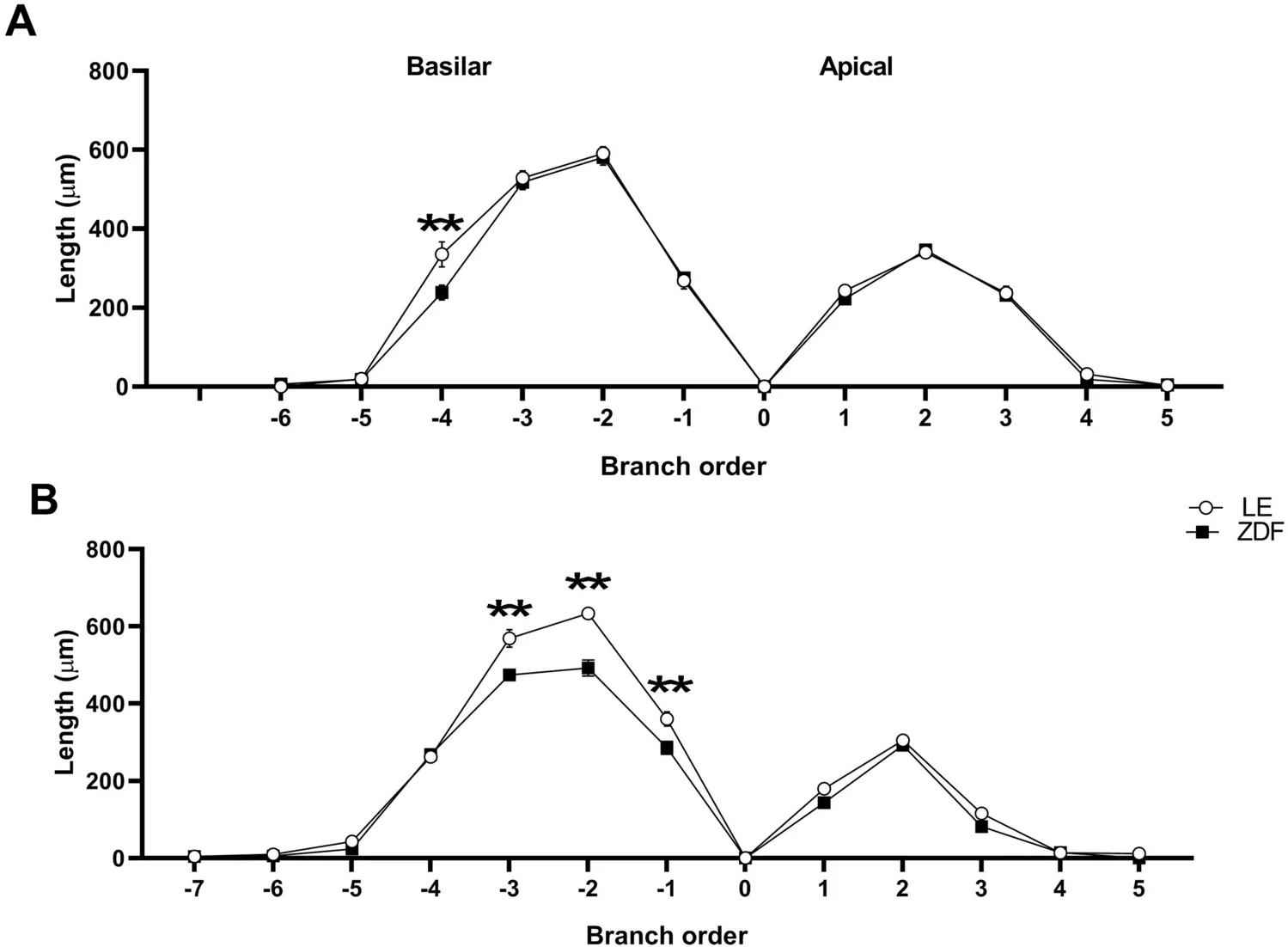
Dendritic length by branch order in M1 pyramidal neurons. **(A)** For female ZDF rats, significant differences were observed at the fourth branch order (−4) in the basilar arbor. **(B)** For male ZDF rats, significant differences were observed in the basilar arbor (−1, −2, and −3 branch order). No changes in dendritic length according to branching order were observed in the apical dendritic arbor of either female or male ZDF rats. The values shown are the mean ± SEM. *N* = 10 per group. ^**^P < 0.001

Photomicrographs of neurons in M1 of LE and ZDF rats are shown in Fig. 5. The results of the two dendritic arbors are shown in a single graph, where negative and positive numbers in the *X*-axis represent the data of the basilar and apical arbors, respectively. Female ZDF rats, compared with female LE rats, exhibited a decrease in the dendritic length of the fourth branch order (−4) in the basilar arbor of M1 neurons, without differences in dendritic length by branch order in the apical arbor (Fig. 6A; Table 4, row A). In the case of male ZDF rats, we observed a decrease in the dendritic length of the first (−1), second (−2), and third (−3) branch orders in the basilar arbor, compared with male LE rats; no differences were observed in the apical arbor between male ZDF and LE rats (Fig. 6B; Table 4, row B).

**Table 4:** Statistical data from the two-way ANOVA applied to the parameters measured in the dendritic arbors of layer V pyramidal neurons in the primary motor cortex (M1)

		Interaction			Sex			Strain		
	Variable	F	*P*	Total variation (%)	F	*P*	Total variation (%)	F	*P*	Total variation (%)
A	Branch order—female	2.336 (13, 250)	0.0060	0.4495	562.34 (13, 250)	< 0.0001	95.65	4.537 (1, 250)	0.0342	0.08121
B	Branch order—male	11.39 (12, 234)	< 0.0001	1.195	916.0 (12, 234)	< 0.0001	96.08	77.64 (1, 234)	< 0.0001	0.6787
C	Basilar dendritic length	0.8858 (1, 36)	0.3529	0.9765	16.75 (1, 36)	0.0002	18.47	37.08 (1, 36)	< 0.0001	40.87
D	Apical dendritic length	0.1455 (1, 36)	0.7051	0.2566	13.21 (1, 36)	0.0009	23.29	7.352 (1, 36)	0.102	12.97
E	Basilar dendritic spine density	0.1162 (1, 36)	0.0002	0.1455	17.73 (1, 36)	0.0002	22.20	26.02 (1, 36)	< 0.0001	32.58
F	Apical dendritic spine density	2.000 (1, 36)	0.1659	1.917	7.548 (1, 36)	0.0093	7.236	58.77 (1, 36)	< 0.0001	56.34

Numbers shown in parentheses represent the degrees of freedom

### Total dendritic length

**Fig. 7:**
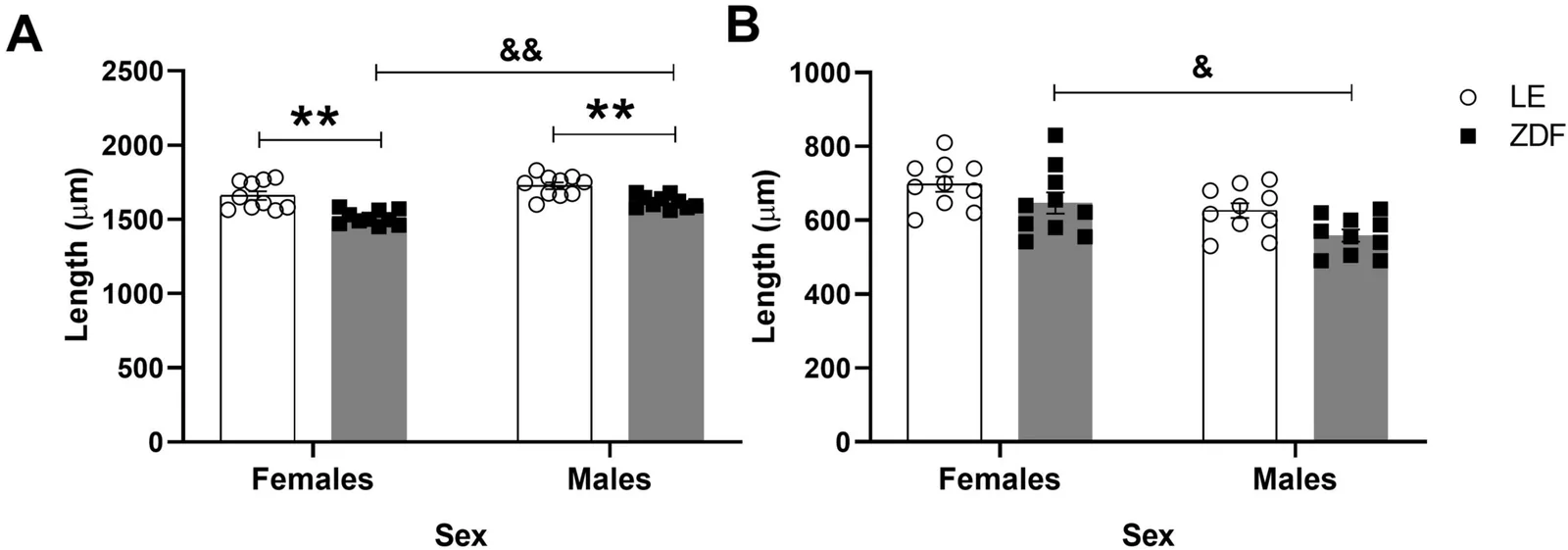
Dendritic length of M1 pyramidal neurons. **(A)** Basilar arbor. There were differences between ZDF rats and their respective control groups, with the difference being greater in female ZDF rats. **(B)** Apical arbor. Dendritic length was shorter in male ZDF rats compared with female ZDF rats. The values shown are the mean ± SEM. *N* = 10 per group. ^**^P < 0.001, *P* < 0.05, and *P* < 0.001

There was a significant decrease in the total basilar dendritic length of M1 neurons in both female and male ZDF rats compared with their respective control groups (Fig. 7A; Table 4, row C). We also found significant differences in the total dendritic length of both arbors between female and male ZDF rats, where M1 neurons of male rats showed longer basilar (*P* = 0.0056) and shorter apical (*P* = 0.0355) dendritic length (Fig. 7B; Table 4, row D).

### Dendritic spine density

**Fig. 8:**
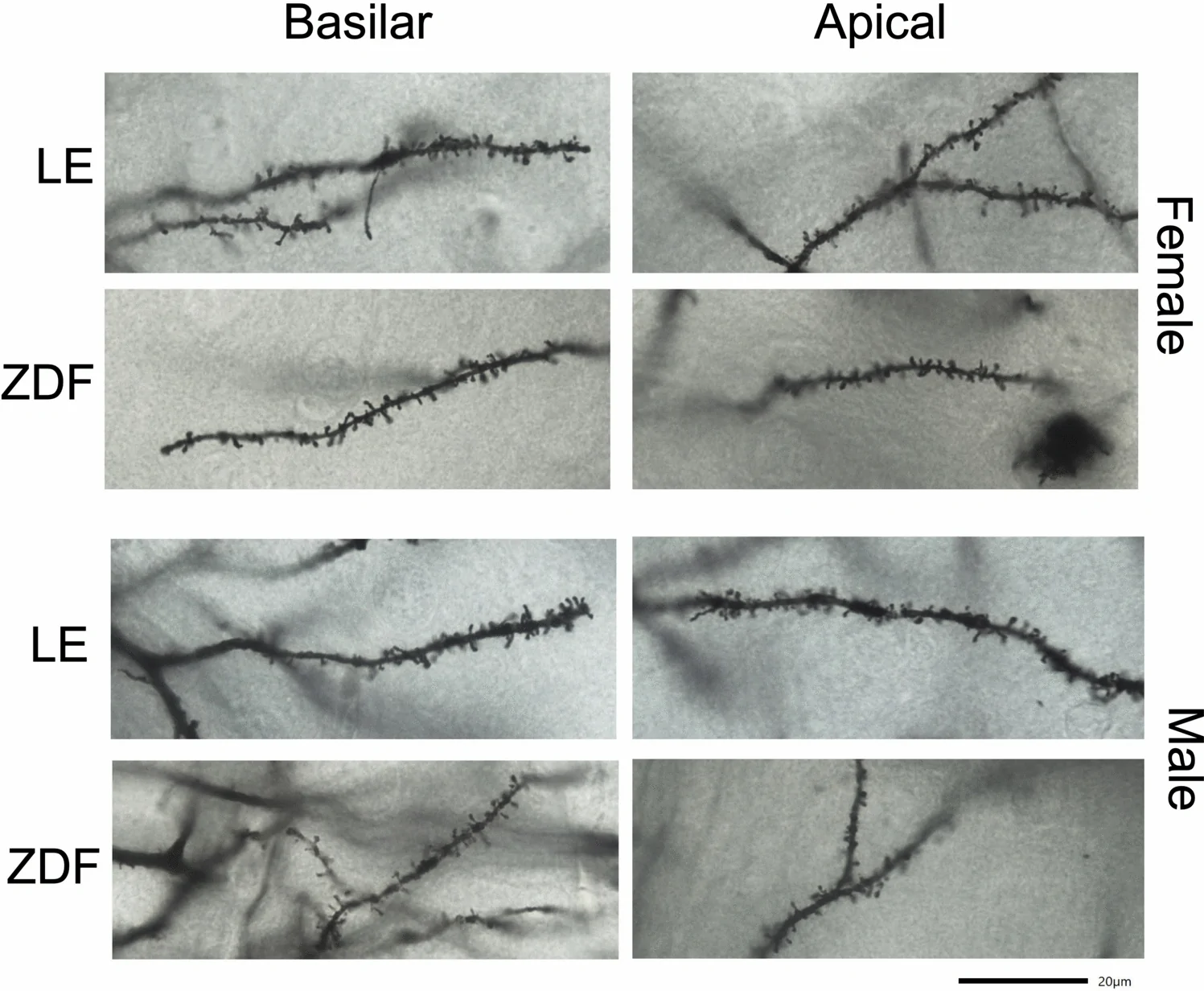
Example of dendritic segments of neurons in M1 in female and male rats. Photomicrographs illustrating Golgi–Cox impregnation of basilar and apical segments of dendrites

**Fig. 9:**
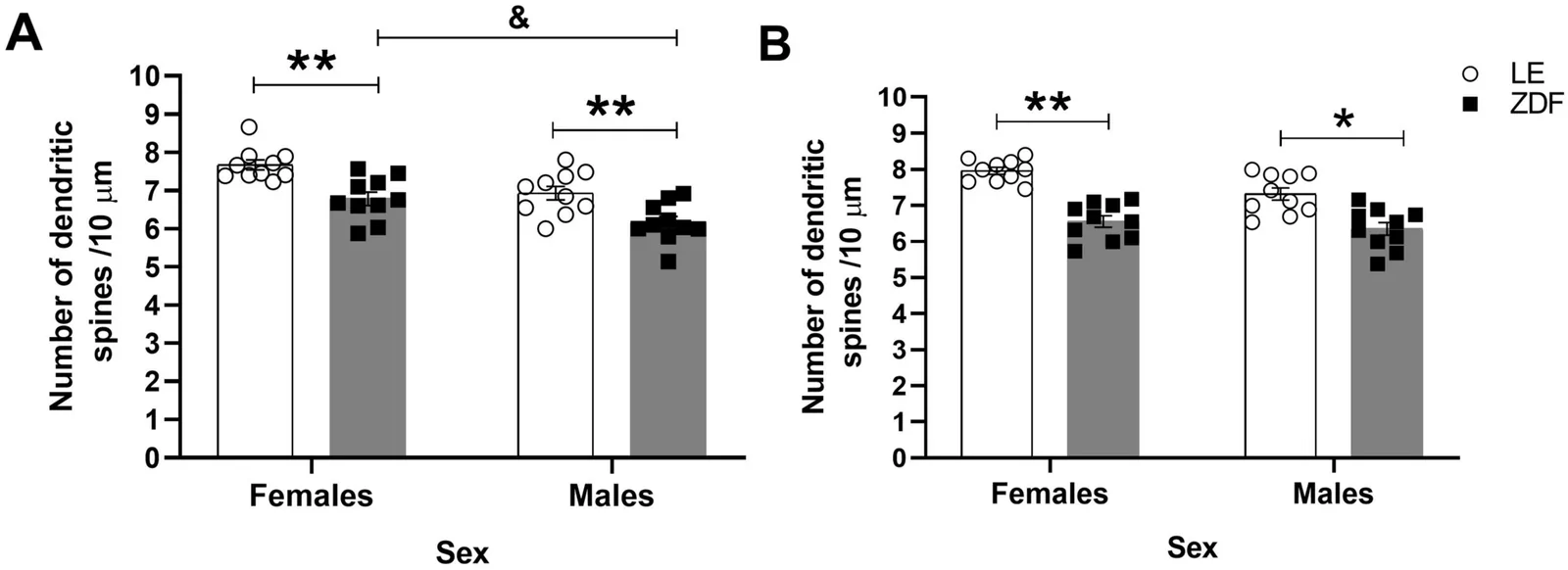
Dendritic spine density. **(A)** Basilar dendritic arbor. A significant decrease was observed in ZDF rats compared with LE rats, which was greater in male ZDF rats. **(B)** Apical dendritic arbor. A significant decrease was observed between ZDF rats and their respective control groups. The values shown are the mean ± SEM. *N* = 10 per group. ^**^P < 0.001 and *P* < 0.05

Photomicrographs of dendritic segments of neurons in M1 of LE and ZDF rats are shown in Fig. 8. Both female and male ZDF rats showed lower dendritic spine density in both the basilar (Fig. 9A; Table 4, row E) and apical arbors (Fig. 9B; Table 4, row F) compared with their respective control groups. In addition, dendritic spine density in the basilar arbor of M1 neurons was significantly lower in male ZDF rats compared with female ZDF rats (Fig. 9A).

Female ZDF rats exhibited reductions in the mean values of dendritic length and dendritic spine density ranging from 7.30% to 17.63%, whereas male ZDF rats showed reductions in the same variables ranging from 6.27% to 13.20% (Table 5).

**Table 5:** Mean differences and percentage reduction in dendritic length and dendritic spine density of the basilar and apical dendritic arbors of M1 neurons in LE and ZDF rats. Female rats exhibited a greater percentage reduction in three of the four neuronal variables analyzed. The data are presented graphically in Figs. 6 and 7, respectively

	Females				Males			
	LE	ZDF	Difference in averages	% reduction	LE	ZDF	Difference in averages	% reduction
Basilar total dendritic length	1659.2 (± 29.28)	1511.3 (± 15.089)	147.9	8.91	1725.5 (± 22.39)	1617.2 (± 13.561)	108.3	6.27
Apical total dendritic length	697.7 (± 20.40)	646.7 (± 28.906)	51.0	7.30	626.5 (± 19.891)	558.8 (± 16.404)	67.70	10.80
Basilar dendritic spine density	7.671 (± 0.131)	6.785 (± 0.176)	0.8860	11.54	6.93 (± 0.176)	6.155 (± 0.163)	0.7750	11.18
Apical dendritic spine density	7.957 (± 0.098)	6.554 (± 0.159)	1.403	17.63	7.314 (± 0.172)	6.348 (± 0.177)	0.9660	13.20

## Discussion

Our results confirm that both female and male ZDF rats included in the present study were obese and had elevated triglyceride and cholesterol levels. ZDF rats of both sexes also exhibited smaller basilar dendritic arbors and lower basilar and apical dendritic spine density in M1 neurons. In addition, male ZDF rats, unlike ZDF females, were hyperglycemic and did not show changes in exploratory activity in the open field.

As observed in adult ZDF rats (4–8 months old), female rats in the present study showed increased body weight (Gale et al. 1981; Bravo-Durán et al. 2021), which indicates obesity according to the Lee index (Lee 1929; Novelli et al. 2007; Arika et al. 2019). We also observed differences in body size related to sex, as male ZDF rats were smaller than male LE rats, whereas female body size was similar between ZDF and LE rats. Obese male ZDF rats aged 7–11 weeks have been shown to exhibit significantly lower growth hormone levels (Finkelstein et al. 1986; DonCarlos et al. 1988; De Lugo and Jurado 2006), as well as reduced tibial and femoral length (Shapira et al. 1980). However, the increase in abdominal circumference observed in both female and male ZDF rats may be related to the hyperphagia that these animals exhibit from an early age (Zucker and Zucker 1961), as well as the development of hyperplasia and adipocyte hypertrophy (Johnson et al. 1971).

In addition to obesity, and consistent with what has been previously reported (Gades et al. 1999; Manuel-Sánchez et al. 2022; Utrilla-Ramos et al. 2024; Bravo-Durán et al. 2021), both male and female ZDF rats in the present study showed high triglyceride and cholesterol concentrations, which have been associated with an increase in lipoprotein lipase activity, leading to higher very-low-density lipoprotein (VLDL) levels associated with increased triglyceride concentrations. Female rats have been reported to exhibit a high content of nonesterified cholesterol associated with VLDL, while male rats show a high content of esters of cholesterol with high-density lipoprotein (HDL) (Fried et al. 1991; Aleixandre de Artiñano and Miguel-Castro 2009). Unlike dyslipidemia, differences in blood glucose levels have been reported in relation to sex. Female ZDF rats in the present study did not show changes in glucose concentration, as observed in previous studies (Chaudhary et al. 2021; Jönsson et al. 2018). However, this result disagrees with a study by Triscari et al. (1979) that showed that 2–3-month-old ZDF rats are hyperglycemic. This metabolic condition appears during the first month of life in male ZDF rats (Hempe et al. 2012; Guzzardi et al. 2022), as confirmed by our results, due to the absence of the glucose-lowering action of leptin (D’souza et al. 2017), insulin resistance (Unger, 1997), and hepatic glucose overproduction (Guzzardi et al. 2022).

In general, the results of the open-field test, showing differences between female and male rats, support the presence of sexual dimorphism in exploratory activity; thus, males and females explore the environment differently (Blizard et al. 1975). Namely, female rats are hyperactive compared with male rats, as they spend more time moving and travel a greater distance (Gould 2009; Tropp and Markus 2001). However, our results indicate that, compared with LE rats, female ZDF rats exhibit reduced exploratory behavior and locomotor activity, a finding consistent with previous reports in male ZDF rats aged 12–20 weeks (Tomassoni et al. 2020). No differences between sexes were observed in ZDF rats, since ZDF females spent the same time moving and traveled the same distance as ZDF males. These findings suggest that obesity has an early effect on locomotor activity in female ZDF rats, which should be examined in future studies to determine whether this effect is attributable to impaired leptin signaling. In this regard, Faber et al. (2021) silenced leptin receptor signaling in the dorsomedial hypothalamic nucleus and reported a reduction in locomotor activity in both female and male mice.

Previous studies investigating dendritic arborization and dendritic spine density in ZDF rats have included animals of only one sex (Bravo-Durán et al. 2021; Manuel-Sánchez et al. 2022; Utrilla-Ramos et al. 2024). In contrast, the present study included both female and male animals, allowing us to demonstrate that ZDF rats of both sexes exhibit alterations in dendritic arborization and dendritic spine density in M1 neurons. Notably, these changes were more pronounced in female ZDF rats, suggesting the presence of sex-related differences. It should also be noted that, although relatively few studies have reported specific measurements of dendritic length and dendritic spine density, the values obtained in both LE and ZDF rats in the present study are comparable with those previously reported for the sensorimotor cortex (apical dendritic length of approximately 700 μm and 0.8–0.9 spines/μm in both dendritic arbors) and for the motor cortex (0.53 ± 0.05 spines/μm in the apical dendritic arbor) (Petit et al. 1988; Davidson et al. 2020).

Our results on dendritic length and dendritic spine density in M1 neurons are consistent with previous reports showing structural alterations in adult female and male ZDF rats, including changes in dendritic arborization of neurons in the ventromedial hypothalamic nucleus, hippocampus, and medial prefrontal cortex. These findings may be related to the observations of O’Malley et al. (2007), who reported that rodents with impaired leptin signaling exhibit reduced hippocampal synaptic plasticity associated with actin filament remodeling. However, future studies should investigate leptin signaling pathways in the brains of ZDF rats to determine whether alterations in this system contribute to the dendritic changes observed in the present study.

However, previous studies conducted in adult rats with diet-induced obesity have shown that female rats with reduced estradiol levels following ovariectomy exhibit metabolic disturbances, reduced hippocampal dendritic arborization, and depression-like behaviors (Sripetchwandee et al. 2019; Boldarine et al. 2019). Similarly, orchiectomized male rats with low testosterone levels display impaired hippocampal plasticity and cognitive dysfunction (Pintana et al. 2016). Nevertheless, previous studies have reported that obese Zucker rats do not exhibit alterations in estradiol or testosterone concentrations during early postnatal development (Withyachumnarnkul and Edmonds 1982; Marín-Bivens and Olster 1997). Therefore, it is reasonable to suggest that the changes observed in the variables measured in the present study are primarily attributable to obesity and to the organizational effects of gonadal hormones acting during the neonatal period, rather than to the activational effects of hormones during puberty or adulthood, as proposed by Darling et al. (2020).

Taken together, we suggest that the reductions in dendritic length and dendritic spine density observed in layer V pyramidal neurons of the primary motor cortex of adolescent ZDF rats may represent structural alterations that impair the stabilization and strengthening of newly formed dendritic spines associated with motor learning (Clark et al. 2018; Peters et al. 2017). Furthermore, these changes may compromise the establishment of the structural substrate required for the storage and spatial organization of motor memory (Fu et al. 2012). Consequently, affected individuals may exhibit limitations in the acquisition, consolidation, and refinement of motor skills.

Finally, it is important to note that the square open-field apparatus may limit the precision of distance-traveled measurements. In addition, correlation analyses (data not shown) between the behavioral variables and dendritic length or dendritic spine density did not reveal any significant associations. Therefore, further studies are needed to identify the factors underlying the reduction in locomotor activity observed in female rats.

## Data Availability

All data generated and analyzed in this study have been included in this published article and are available upon request.
